# Luciferase-Expressing *Leishmania infantum* Allows the Monitoring of Amastigote Population Size, *In Vivo*, *Ex Vivo* and *In Vitro*

**DOI:** 10.1371/journal.pntd.0001323

**Published:** 2011-09-13

**Authors:** Grégory Michel, Bernard Ferrua, Thierry Lang, Madhavi P. Maddugoda, Patrick Munro, Christelle Pomares, Emmanuel Lemichez, Pierre Marty

**Affiliations:** 1 Université de Nice-Sophia Antipolis, Faculté de Médecine, Nice, France; 2 Université de la Méditerranée, Marseille, France; 3 Bâtiment Universitaire Archimed, INSERM U895, C3M, BP 2 3194, Nice, France; 4 Centre Hospitalier Universitaire de Nice, Laboratoire de Parasitologie-Mycologie, Nice, France; 5 Institut Pasteur, Département de Parasitologie, Immunophysiologie et Parasitisme Intracellulaire, Paris, France; Institut Pasteur, France

## Abstract

Here we engineered transgenic *Leishmania infantum* that express luciferase, the objectives being to more easily monitor in real time their establishment either in BALB/c mice—the liver and spleen being mainly studied—or *in vitro*. Whatever stationary phase *L. infantum* promastigotes population—wild type or engineered to express luciferase—the parasite burden was similar in the liver and the spleen at day 30 post the intravenous inoculation of BALB/c mice. Imaging of *L. infantum* hosting BALB/C mice provided sensitivity in the range of 20,000 to 40,000 amastigotes/mg tissue, two tissues—liver and spleen—being monitored. Once sampled and processed *ex vivo* for their luciferin-dependent bioluminescence the threshold sensitivity was shown to range from 1,000 to 6,000 amastigotes/mg tissue. This model further proved to be valuable for *in vivo* measurement of the efficiency of drugs such as miltefosine and may, therefore, additionally be used to evaluate vaccine-induced protection.

## Author Summary

*Leishmania infantum*/*L. chagasi* parasites are inoculated in the skin of mammals by sand flies. Though most often these *L. infantum*-mammal interactions are asymptomatic, they can proceed, in some individuals, to a systemic disease known as visceral leishmaniasis. If left untreated this disease is fatal. The lack of protective or curative vaccines and the limited number of parasite-targeting drugs were incentive to set up experimental conditions that could allow easy monitoring of the fluctuation of the population size of parasites in living laboratory animals. Thus, in the present report, we depict two distinct readout assays that rely on a population of *L. infantum* we genetically engineered for stably expressing the firefly luciferase gene. These transgenic parasites were either inoculated to BALB/c mice or added to a culture of monocytic cells. Post intravenous inoculation, BALB/c mice were imaged over time, with special attention being given to the liver and the spleen. The sensitivity of this technique ranged from 20,000 to 40,000 parasites/mg of tissue and from 1,000 to 6,000 parasites/mg tissue, for *in vivo* and *ex vivo* measurements, respectively. Though preliminary, the data, relying on monocytic cells, are promising for further *in vitro* screening of small compound libraries.

## Introduction

*Leishmania* are obligate intracellular dimorphic protozoan parasites that cause a broad spectrum of clinical diseases in mammalian hosts. Visceral leishmaniasis, due to *L. infantum*, is endemic in the mediterranean basin (Mediterranean Visceral Leishmaniasis, MVL) and is a fatal disease, if untreated. To date, no efficient vaccine exists against human MVL and therapeutic options for managing MVL are limited with significant toxicity in some cases.

Exploring novel molecules for use as leishmanicidal drugs or vaccines necessitates experimental models such as *in vitro* culture of mouse or human-derived macrophages or laboratory susceptible mice. The standard method for monitoring infection in the mouse model is based on the estimation of parasite loads in target organs such as liver, spleen, or lymph nodes by microscope examination of touch imprints or smears. Alternatively, limiting culture dilution or qPCR amplification of parasite DNA is performed [1]. These techniques are however cumbersome and require large groups of mice to be euthanized to follow the efficiency of leishmanicidal drugs or time-course efficacy of vaccines against MVL. To overcome these drawbacks, a few real time monitoring methods, using reporter genes encoding GFP or firefly luciferase, have been developed for *in vitro* drug screening [2]–[7] or *in vivo* individual follow-up of *Leishmania* infection in the mouse model [8]–[13]. Most *in vivo* studies have focused on dermotropic species. Only a limited number of experiments have been conducted concerning visceral species (*L. donovani and L. chagasi*), which target deep organs [10], [13]. In particular, no complete study using transgenic *L. infantum* has until now been reported.

In this paper, we have engineered transgenic *L. infantum* stably expressing luciferase. We then aimed to assess their usefulness for monitoring - *in vivo* and *ex vivo* - *L. infantum* biomass overtime in liver and spleen of BALB/c mice inoculated with a high parasite dose. Particular attention has been paid to the suitability - *in vitro* and *in vivo* - of recombinant *L. infantum*-expressing luciferase for screening the efficiency of leishmanicidal drugs such as miltefosine.

## Materials and Methods

### Mice and ethics statement

Six to eight week-old female BALB/c mice were purchased from Charles River (France). Mice were maintained and handled according to the regulations of the European Union, the French Ministry of Agriculture and to FELASA (the Federation of Laboratory Animal Science Associations) recommendations. Experiments were approved by the ethics committee of the Nice School of Medicine, France (Protocol number: 2010-45).

### Promastigote culture

*L. infantum* MON-1 (MHOM/FR/94/LPN101), was isolated from a patient with MVL contracted in the Nice area (South of France). *L. infantum* promastigotes were routinely grown at 26°C in M199 medium supplemented with adenosine 0.1 mM, biotin 1 µg/ml, bovine hemin 5 µg/ml, streptomycin 100 µg/ml, penicillin 100 U/ml, 2 µg/ml biopterin, L-glutamine 2 mM, folic acid 10 µg/ml and 10% fetal calf serum (culture medium) [14].

### Generation of recombinant *L. infantum*-expressing reporter genes

*L. infantum* clones encoding firefly luciferase were generated as previously described [8]. Briefly, the 1.66 kb coding region of firefly luciferase was cloned into the *Leishmania* expression vector pF4x1.HYG, with marker gene for selection with hygromycin B. Following linearization by SwaI restriction digest, an insert containing 18S rRNA flanked with the luciferase gene and hygromycin, was prepared for integration into the 18S rRNA locus of the nuclear DNA of *L. infantum*. *L. infantum* promastigotes (0.5×10^8^), in exponential growth phase, were suspended in 0.5 ml of cytomix buffer (25 mM HEPES pH 7.5, 0.15 mM CaCl_2_,120 mM KCl,10 mM KH_2_PO_4_, 2 mM EDTA, 5 mM MgCl_2_). Transfections were performed by electroporation (Gene Pulser X cell System, Biorad) using 3 µg of DNA inserts with the following conditions: 25 µF, 1600 v, 9 ms in 4 mm cuvette. Following electroporation, transfected parasites were cultured in complete culture medium and plated on semi-solid medium containing 100 µg/ml of hygromycin B. *L. infantum* colonies were collected, expanded in culture medium with hygromycin, aliquoted and frozen in liquid nitrogen in 90% fetal calf serum with 10% DMSO until use. Before inoculation experiments, a single promastigote clone termed LUC-parasite was passaged twice in M199 medium containing hygromycin. Occasionally, to maintain virulence, the LUC-parasite was injected by intra-peritoneal (IP) route to BALB/c mice and two months later, spleen or liver was collected and cultured as a source of promastigotes.

### Inoculation with LUC-parasites or wild type parasites *in vivo* and quantification of parasite loads

Variable inocula of the stationary phase LUC-parasites or wild type (WT) promastigotes (ranging from 0.12×10^8^ to 3×10^8^ parasites) in stationary phase of growth, were injected by intravenous (IV) route to groups of 4 of 6–8 week-old female BALB/c mice. One month later, the animals were euthanized by CO_2_ and spleens and livers were collected. Spleen and liver aliquots were homogenized at 100 mg/ml in PBS containing 1% Nonidet P40 and a protease inhibitor cocktail (Roche), and parasite loads were quantified by an ELISA-based method as previously described [15], [16]. For follow-up studies, mice were infected as above, and at different time-points imaging was performed. Occasionally, mice were infected by the intraperitoneal route (IP) with 500×10^6^ stationary phase LUC-parasites and regularly imaged.

### Bioluminescence imaging of *L. infantum* infected BALB/c mice

Mice infected with LUC-parasites were periodically imaged using the Photon Imager (Biospace Lab, France) system as follows: mice were administered with luciferin (Caliper life science, France) at 300 mg/kg by IP route, and within 10 min, the animals were anesthetized in 5% isoflurane/1L O_2_.min^−1^ atmosphere. The animals were then placed in the imaging chamber of the Photon Imager and anesthetisis was maintained using 2.0% isoflurane/ 0.2 L O_2_ per mouse min-1 atmosphere. Acquisition of emitted photons, with a charge-coupled device camera, was monitored over a 20 min period in previously defined regions of interest (ROI) that delimited the surface of analysis. To standardize imaging, and to allow comparison between mice, the images presented in the figures correspond to an acquisition of 1 min duration, taken once luminescence plateaued. In some experiments, bioluminescence imaging (BLI) localization of transgenic luciferase expressing *L. infantum* amastigotes to mouse liver, spleen or other sites was confirmed by reimaging mouse after dissection.

### *In vitro* analysis of luminescence activity and quantitation of parasite loads *ex vivo*

*In vitro* luminescence activity was measured using the Luciferase Assay System E1501 (Promega). To quantify *ex vivo* parasite burdens by luminescence, Nonidet P40 extracts of spleen or liver, prepared as above, were serially diluted with the reporter lysis buffer. Luminescence was quantified from 10 µl aliquot (1 mg tissue) using a luminometer (Centro LB 960 Berthold Technologies, Germany). To compare luciferase activity of promastigote and amastigote forms, the exponentially growing LUC-promastigote clone, and spleen or liver aliquots from infected mice, were extracted with NP 40 buffer and parasites number was quantified by ELISA. Detergent extracts were serially diluted and luminescence activity was analysed as above.

### *In vitro* infection of monocytic cells with luciferase clone for drug screening

The THP-1 cell line (ATCC TIB-202) was routinely grown in RPMI 1640 medium containing 10% fetal calf serum and differentiated overnight at 10^6^/ml density with 10 ng/ml of phorbol myristate acetate (PMA) in 96-well microtiter plate. Wells were then washed with RPMI and fresh complete medium was added. PMA-differentiated THP-1 cells were infected with stationary phase LUC-parasites, at parasite to THP-1 cell ratio of 10∶1, for 3 h at 37°C. Free parasites were aspirated off and the plate was incubated for 48 h in culture medium for amastigote transformation. Miltefosine (hexadecylphosphocholine, Sigma) was added at concentrations ranging from 10^−8^ to 10^−4^ M and within 48 h the plate was washed and the wells were extracted using 50 µl of reporter lysis buffer. Luminescence activity was quantified from 20 µl aliquot as described above. Cell viability was evaluated using Trypan blue exclusion.

## Results

### Infectivity of luciferase *L. infantum* and *in vivo* and *ex vivo* evaluation of parasite burdens using bioluminescence

**Figure 1:**
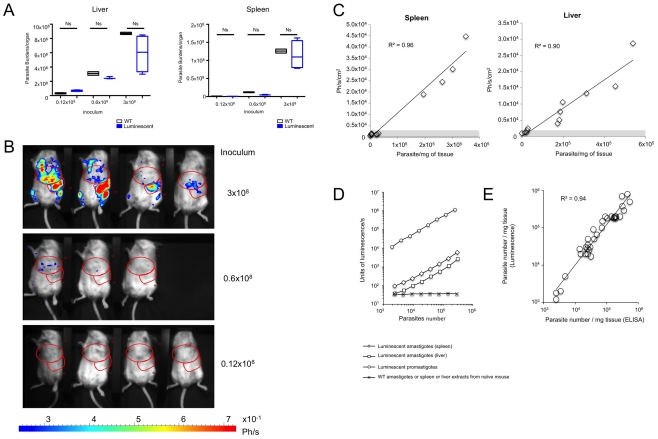
Infectivity of LUC-parasites and sensitivity of bioluminescence *in vivo* and *ex vivo*. A. *Infectivity of Luc-parasites as compared to L. infantum WT parasites*. Both luciferase transgenic and wild type *L. infantum* stationary phase promastigotes establish themselves in BALB/c mice. Groups of 4 BALB/c mice were given either 0.12×10^8^ to 3×10^8^ stationary phase luciferase-transgenic or wild type *L. infantum*. One month later, mice were imaged, the bioluminescence being recorded before their sacrifice. *L. infantum* burdens were estimated by ELISA in the liver and the spleen. Burdens were calculated as parasite/mg organ x organ weight (in mg). Data representative of three experiments are presented as box whisker plots. For statistical analysis Man Whitney Wilcoxon test was performed and did not show any significant difference between parasite loads of mice inoculated with WT or luciferase parasites; ns =  not-significant. B. *Bioimaging of BALB/c mice one month post the IV inoculation of luciferase transgenic L. infantum*. Mice inoculated with LUC-parasite and with parasite loads depicted in figure 1A, were given luciferin via IP. The photon emission was recorded once the anesthetized mice were deposited in the imaging chamber of the Photon Imager. Red zones are ROIs that delineate the liver or spleen. C. *Sensitivity of BLI.* Luminescence (photon/s/cm^2^) was recorded in ROIs corresponding to liver and spleen of the 11 mice infected with LUC-parasites depicted in figure 1A. Luminescence recorded in ROIs was plotted versus parasite density measured by ELISA as described in figure 1A to generate regression curves. The threshold sensitively of BLI calculated as three times luminescence of naïve mice (indicated by the gray zone) was 20,000 to 40,000 parasites/mg for spleen and liver respectively. D. *Ex-vivo quantification of parasite loads by bioluminescence*. Luminescence of equal numbers of the LUC-parasite under exponentially growing promastigote form or liver and spleen amastigotes was analyzed using a luminometer. Equal numbers of liver and spleen amastigotes from mice infected with WT promastigotes were used as specificity control. Sensitivity of *ex vivo* analysis of parasite density by bioluminescence (1,000 to 6,000 amastigotes/mg for spleen and liver respectively) was calculated as parasite numbers corresponding to twice luminescence background values. E. *Accuracy of ex vivo bioluminescence analysis for estimation of parasite density*. Liver and spleen samples from 18 IV infected BALB/c mice were detergent extracted and assayed both by ELISA and bioluminescence. Values generated by both techniques were plotted to generate regression curves.

In order to assess the infectivity of the *L. infantum* luciferase parasites (LUC-parasite), as well as the usefulness of bioluminescence for the monitoring of parasite proliferation in target organs, BALB/c mice were inoculated by IV route with various inocula of the stationary phase LUC-parasites or WT parasites. One month following inoculation, when generally both liver and spleen are infected, mice were imaged and sacrificed. Detergent extracts from liver or spleen were prepared for parasite quantification by ELISA or bioluminescence analysis *ex vivo*. Figure 1A shows that mice inoculated with increasing numbers of the LUC-parasite or WT parasites exhibited increasing and similar spleen and liver parasite burdens. This indicates that the selected LUC-parasite exhibits an infectivity identical to that of WT parasites (Fig. 1A). Identical results were obtained with another LUC-parasite (data not shown). In order to evaluate the threshold sensitivity of BLI, LUC-parasite infected mice were imaged and the luminescence (expressed as photons/s/cm^2^) present in a ROI corresponding to liver or spleen was recorded. Despite variable infection levels in mice infected with the same parasite dose, imaging of bioluminescence signals confirmed the parasite dose-dependency of the infection measured by ELISA (Fig. 1A). Luminescence measured in the ROI and express as photon/s/cm^2^, was directly proportional to parasite density, and the generated regression curves were used for estimating the threshold sensitivity of BLI for both organs (Fig. 1B). The threshold sensitivity of BLI was calculated as luminescence three times stronger than background luminescence of naïve mice, which exhibit the same background values as non-bioluminescent parasite infected mice. In our hands, threshold sensitivity was around 20,000 and 40,000 parasites per mg spleen and liver, respectively (above the grey zone of Fig. 1C).

We next assessed the possibility of measuring parasite loads by *ex vivo* analysis of organ luminescence. *L. infantum* parasites are present in the host as intracellular amastigotes, and are the form targeted by drugs and vaccines. As these amastigote forms generally display a lower metabolic activity than exponentially growing promastigotes we compared *in vitro* luciferase activity of both parasite forms. Figure 1D shows the dose-response curves drawn from luciferase parasites under promastigote or spleen and liver amastigote forms. As expected, luciferase activity of amastigotes was notably lower than that of exponentially growing promastigotes with threshold sensitivities (calculated as twice the background value) at around 10, 1,000 and 6,000 parasites for metacyclic promastigotes, spleen and liver amastigotes, respectively (Fig. 1D). The specificity of *ex vivo* analysis was confirmed by the lack of luminescence activity of equal numbers of spleen and liver WT amastigotes. Analysis of luminescence *ex vivo* thus facilitates a rapid and simple mean for quantitating parasite burdens in mouse organs provided a relatively high infection level is present. Finally, parasite density of samples taken from 36 livers or spleens were analysed both by luminescence analysis and ELISA. Strong correlation between the two methods verified the reliability of the bioluminescence assay (Fig. 1E).

### Monitoring of *L. infantum* infection and *in vivo* drug screening by bioluminescence imaging

**Figure 2:**
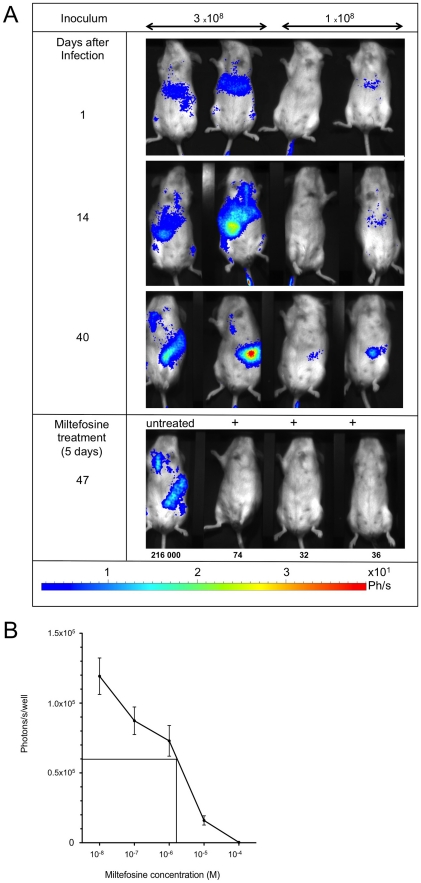
Monitoring of *L. infantum* infection using BLI and application for leishmanicidal drug screening. A. *Follow up of infection by BLI and drug screening in vivo*. Groups of 2 BALB/c mice were IV infected with 1×10^8^ or 3×10^8^ of stationary phase LUC-parasites and animals were imaged at the indicated times. BLI of mice shows the typical liver infection followed by spleen parasite colonization. By day 40, 3 mice with different levels of spleen infection were treated with miltefosine during 5 days (+) and reimaged 2 days later. Miltefosine efficacy was illustrated by the loss of luminescence signal compared to a control non treated mouse. Efficacy was confirmed by parasite counts (number of parasites/mg organ) measured by ELISA following dissection (indicated below each mouse tested). The illustration is representative of three different experiments. B. *In vitro drug screening*. PMA-differentiated THP-1 cells in 96 well microplates were incubated for 3 h with stationary phase LUC-parasites at a parasite-to-THP-1 cell ratio of 10∶1. After 48 h incubation, miltefosine at concentrations ranging from 10^−4^ to 10^−8^ M were delivered. 48 h later, the plate was washed and wells were lysed with 50 µl of reporter lysis buffer. 20 µl of supernatant was assayed for luminescence activity and showed a clear negative correlation between the drug concentration and luminescence of parasites. IC 50 was measured at 1.5 µM. Cell viability measured by trypan blue exclusion was 83% and 96% for miltefosine concentration of 100 µM and 10 µM, respectively. The dose dependency of miltefosin efficacy on parasite killing is representative of 3 different experiments.

BALB/c mice inoculated with *L. infantum* WT parasites generally display a liver infection episode, which peaks 1–2 months post inoculation and is partially self-resolved because of granuloma formation [17]. Partial liver clearance is followed by progressive and chronic spleen infection with a concomitant destruction of spleen architecture [17]. We used this model of infection to assess the usefulness of bioluminescence to follow this process. For this, BALB/c mice were intravenously infected with 3×10^8^ and 1×10^8^ stationary phase promastigotes of the LUC-parasite, and at different time-points, the spread of infection was recorded following IP injection of the luciferin substrate. Time-course infection followed by BLI (Fig. 2A) revealed the classical transitory hepatic episode (day 14) followed by parasite spleen colonization (day 40) (Fig. 2A). Of note, in the two heavily infected mice (3×10^8^), inguinal and mediastinal lymph nodes were persistently bioluminescent (Fig. 2A). Conversely, bone marrow parasite localisation could not be evidenced.

Next, in order to validate this technique for drug screening purposes, either *in vivo* or *in vitro*, we conducted experiments using miltefosine as a leishmanicidal drug. For *in vivo* testing, infected mice were treated with miltefosine for 5 days (1 mg per mouse per day) and imaged 2 days later. Figure 2A shows that parasite decrease can be readily detected by BLI imaging *in vivo*, as luminescence was undetectable in spleen or lymph nodes following 5 days of treatment (Fig. 2A). This reduction was seen regardless of the infection level before treatment and was confirmed following dissection of mice and measurement of spleen burdens (Fig. 2A). Therefore, parasite clearing by miltefosine in spleen was accompanied by the disappearance of luminescence.

Similarly, luminescence was used *in vitro* to evaluate the efficacy of miltefosine on intracellular amastigotes. PMA-differentiated THP-1 cells were infected with metacyclic LUC-parasites and within 48 h, a time sufficient for amastigote transformation (data not shown), miltefosine was added. 48 h later, THP-1 cells were lysed and luciferase activity was quantified. Figure 2B shows the drug-concentration dependency of amastigote killing by miltefosine (Fig. 2B). The IC _50_ (1.5 µM) was identical to that previously reported [18]. Using this method, drug-concentration dependency of amastigotes killing could be measured (Fig. 2B). This protocol may therefore provide a high throughput method for screening drugs.

### Demonstration of a new potential site of parasite proliferation by bioluminescence imaging

**Figure 3:**
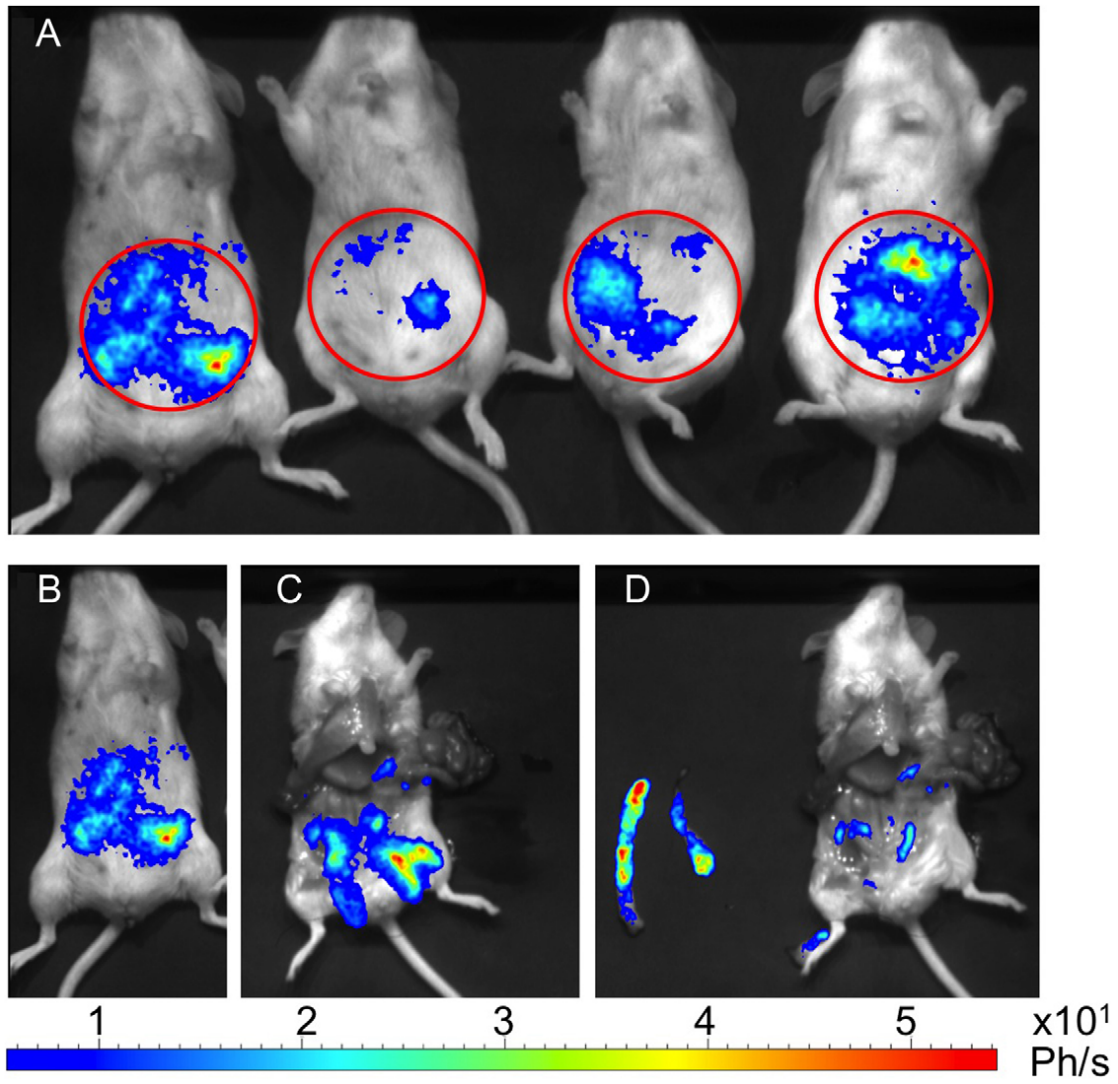
Localization of bioluminescent parasites in intra-abdominal mesenteric fat in BALB/c mice infected by IP route. 4 Mice were infected with 5×10^8^ of LUC-parasites by IP route. Infection was monitored by BLI. On day 40 post infection (Fig 3A), mice showed intraperitoneal parasites localisation by BLI. One representative mouse (Fig 3B) was dissected and the adipose tissue localization detected by bioluminescence was verified *in situ* (Fig 3C) and after removing the adipose tissue and reimaging (Fig 3D). The illustrations are representative of at least three different experiments.

Preliminary experiments showed that BALB/c mice infected via the IP route with 5×10^8^ LUC-parasites displayed hepatic and spleen infection episodes similar to that observed following IV challenge (data not shown). However, infection levels in target organs were generally lower than that observed following infection by the IV route. Nevertheless, by day 40 post inoculation, peritoneum bioluminescence, clearly distinct from spleen bioluminescence (Fig. 3A, 3B), was detectable in nearly all infected mice and was still observable 3 months post inoculation (data not shown). Dissection of mice showed that peritoneal bioluminescent parasites localized to a mass of adipose tissue located above the intestine (Fig. 3C, 3D). Interestingly, BLI and quantitation of amastigotes in adipose tissue indicated that compared to spleen, 20 times more photons per parasite could be recovered from peritoneum amastigotes. Therefore, BLI can be useful to demonstrate unexpected sites of parasite proliferation.

## Discussion

The BALB/c mouse model has been widely used for drug screening and vaccine trials, since therapeutic effects of drugs or vaccine-induced protection are evaluated keeping the contextual influences of the living animal. However, estimation of parasite loads at different time-points in target organs necessitates euthanizing and dissecting animal groups, thus limiting longitudinal studies devoted to the therapy of MVL. In addition, parasite loads are extrapolated from parasite counts obtained on a limited sample size, which is not always representative of the burden occurring in the organ. Finally, spread of parasites to an unexpected site of infection may be missed because the infected tissue is not harvested or analysed.

Recently, the possibility of labelling invasive microorganisms with reporter genes, such as firefly luciferase has provided, the ability to trace the infection dissemination at the tissue/organ level by BLI in animal models. BLI potentially presents many advantages over conventional methods of infection monitoring. BLI technology allows detection of only live, metabolically active cells and because of its non-destructive and non-invasive nature it can be performed repeatedly. BLI thus permits each animal to be used as its own control over time, overcoming the problem of animal-animal variations. Finally, as already reported, by using this technique new microorganism localization can be evidenced [19].

We report here the generation of recombinant luciferase *L. infantum* parasites and their use in tracing parasite dissemination *in vitro* and *in vivo*. Our results show that luciferase transfected parasites are suitable for both *in vitro* and *in vivo* studies. While light emission of the amastigote form was reduced as compared to that of extracellular promastigotes it was sufficient to provide an accurate and rapid estimation of parasite loads *ex vivo* with a sensitivity of around 1 to 6,000 amastigotes/mg tissue, which is sufficient for most applications. Importantly, luciferase parasite clones proved to be also useful tools to measure drug efficacy of miltefosine *in vitro* and *in vivo* at the amastigote stage. Luminescent *L. infantum* parasites may thus represent a high throughput method for leishmanicidal drug testing on different target organs in the context of the whole body.

BLI of BALB/c mice infected with luciferase clones showed that in our conditions, the threshold sensitivity of parasite burdens was around 20×10^3^ to 40×10^3^ parasites/mg for spleen and liver. This indicates that BLI of deep organs requires relatively high parasite loads. In our hands, these infection levels could be easily reached by IV route using high parasite inoculum not enriched in mammal-invasive metacyclic promastigotes. Following IP parasite delivery we noticed a new site of parasite development located in the peritoneum. The demonstration of this unexpected localization, which has not been observed in mice infected by IP with *L. donovani* [8], suggests that this localisation may be species specific. The high photon emission observed in adipose tissue (numbers of photon/parasite) as compared to spleen emphasizes the fact that, due to different local environments, luminescence emitted by different organs cannot be compared but for a given organ it reflects differences in parasite loads. Absorption and scattering of light by overlaying tissues may account for this difference though it is likely that the delivery of luciferin directly into the peritoneum facilitated better photon emission from the peritoneum. Collectively we demonstrate that BLI represents a versatile tool for drug screening *in vitro* or *in vivo* as well as for assessing the potential protectivity of vaccine preparations. More sensitive cameras and/or luciferase with improved catalytic activity or a higher vector expression [20], will undoubtedly improve the performance and ability of BLI of *L. infantum*.
